# Special Issue “Mitochondrial Remodeling and the Targeting Strategies for Disease Treatment”

**DOI:** 10.3390/ijms262311370

**Published:** 2025-11-25

**Authors:** Yong Teng

**Affiliations:** 1 Department of Hematology and Medical Oncology, Winship Cancer Institute, School of Medicine, Emory University, Atlanta, GA 30322, USA; yong.teng@emory.edu; Tel.: +1-(404)712-8514; 2 Wallace H. Coulter Department of Biomedical Engineering, Georgia Institute of Technology & Emory University, Atlanta, GA 30322, USA

Mitochondria are highly dynamic and responsive organelles that perform multifaceted functions depending on the environmental conditions and cellular demands. Beyond their traditional role as the “powerhouses” of the cell, mitochondria actively sense and adapt to metabolic, oxidative, and signaling cues. It is now well established that these organelles undergo profound alterations in their structure, proteome, metabolism, and even genome in response to cellular and environmental stimuli [1,2,3]. As such, mitochondria serve as central hubs for diverse signaling processes that are fundamental to cellular homeostasis, energy production, metabolism, survival, and death.

Mitochondrial remodeling, encompassing structural rearrangement, recycling through mitophagy, and functional reprogramming, is essential for maintaining mitochondrial quality control and ensuring their proper communication with other cellular organelles [1,3,4]. These remodeling processes enable cells to dynamically adapt to physiological changes and to withstand various forms of stress. However, when dysregulated, mitochondrial remodeling can drive pathological states. Emerging evidence links dysregulated mitochondrial dynamics to the development of diverse human diseases, including cancer. Numerous studies have demonstrated that mitochondrial dysfunction drives cancer progression by reshaping metabolic pathways, increasing reactive oxygen species, impairing apoptotic signaling, and enabling tumor cells to adapt and survive in adverse microenvironments [5,6]. Beyond cancer, mitochondrial dysfunction has become a recognized driver of neurodegenerative conditions through impaired bioenergetics, defective mitophagy, and increased oxidative stress [7]. In neurodevelopmental disorders, disruptions in mitochondrial metabolism can alter synaptic maturation and neuronal signaling, thereby influencing early brain development [8]. These insights underscore the broad involvement of mitochondrial biology across neurodegenerative and neurodevelopmental psychiatric conditions and aging-related pathologies.

Despite rapid advances in mitochondrial biology, the mechanisms driving mitochondrial remodeling remain poorly understood, hindering therapeutic development for mitochondria-related diseases. However, recent innovations in high-resolution imaging, multi-omics technologies, and sophisticated genetic and biochemical tools have created unprecedented opportunities to dissect the spatiotemporal regulation of mitochondrial structure and function [3,4,5,6]. These innovations now allow researchers to investigate mitochondrial remodeling with remarkable precision and to uncover how these dynamic processes contribute to disease initiation, progression, and therapeutic response. Motivated by these emerging insights, this Special Issue has been launched to advance our understanding of the role, regulation, and therapeutic targeting of mitochondrial remodeling in human diseases.

In this Special Issue, several studies illuminate how mitochondrial perturbations contribute to pathophysiology and highlight innovative approaches to restore mitochondrial function. Fibrotic diseases exemplify the link between mitochondrial dysfunction and pathological cellular remodeling. Fibroblast-to-myofibroblast transition (FMT), driven primarily by TGF-β signaling, underlies excessive extracellular matrix (ECM) deposition and is associated with metabolic reprogramming toward glycolysis. A featured study demonstrates that the delivery of dextran–triphenylphosphonium (Dex–TPP)-coated mitochondria to TGF-β-stimulated fibroblasts suppresses glycolysis, reduces oxidative stress, and attenuates FMT, illustrating the therapeutic potential of mitochondrial supplementation (contribution 1). Similarly, in idiopathic pulmonary fibrosis (IPF), treatment with the prostacyclin analog treprostinil restored mitochondrial integrity and normalized the expression of key mitochondrial regulators, including PTEN and PINK1 (contribution 2). Treprostinil also enhanced autophagic quality control, providing mechanistic insight into its clinically observed therapeutic benefits (contribution 2). Beyond fibrotic remodeling, mitochondrial dysfunction also drives cardiovascular complications in metabolic disease. In type 2 diabetes (T2D), vascular smooth muscle cells (VSMCs) exhibit impaired mitochondrial Ca^2+^ handling, leading to elevated cytosolic Ca^2+^, sustained Erk1/2 activation, and excessive proliferation (contribution 3). Interventions that restore mitochondrial Ca^2+^ homeostasis or inhibit the mitochondrial permeability transition pore (mPTP) attenuated pathological VSMC growth, identifying mitochondrial Ca^2+^ dynamics as a potential therapeutic target in diabetic vascular disease (contribution 3).

Mitochondrial integrity is likewise crucial in preserving skeletal muscle function. Hibernating mammals provide a striking example: during torpor, muscle contractility and fatigue resistance decline, yet brief interbout arousals restore both, coinciding with increased mitochondrial respiratory chain activity (contribution 4). These findings reveal that periodic mitochondrial reactivation is essential to prevent disuse-induced muscle atrophy, highlighting a physiological mechanism that safeguards tissue integrity. Liver metabolic disorders further underscore the centrality of mitochondria in systemic homeostasis. In metabolic dysfunction-associated steatotic liver disease (MASLD), mitochondrial beta-oxidation is impaired, and mitochondrial DNA methylation contributes to lipid dysregulation. The overexpression of CpG and GpC DNA methyltransferases in mitochondria induced mito-nuclear epigenetic reprogramming, promoting cholestasis, mitochondrial swelling, lipid accumulation, and mitophagy (contribution 5). Complementing this epigenetic perspective, another study shows that the pharmacological targeting of mitochondrial dynamics can protect hepatic function (contribution 6). Specifically, cilnidipine and its derivative 1,4-DHP inhibited pathological Drp1–filamin interactions, reducing lipid droplet accumulation and cytotoxicity in hepatocytes and mouse livers. These findings reveal a promising strategy to preserve mitochondrial lipid homeostasis.

Collectively, these studies underscore mitochondrial dysfunction as a shared and modifiable driver of fibrosis, metabolic disorders, vascular pathology, and muscle disuse atrophy (contribution 1–6). By uncovering the mechanisms underlying mitochondrial impairment and advancing pharmacologic and cellular interventions, this body of work establishes mechanism-driven, mitochondria-targeted strategies as a fertile avenue for novel therapies across diverse disease contexts. We hope that the findings and perspectives highlighted in this Special Issue will stimulate new ideas, encourage integrative collaborations across scientific fields, and drive the next generation of research aimed at harnessing mitochondrial remodeling as a therapeutic strategy for human disease.

## References

[1] Chandel N.S. (2018). Mitochondria: Back to the future. Nat. Rev. Mol. Cell Biol..

[2] Monzel A.S., Enríquez J.A., Picard M. (2023). Multifaceted mitochondria: Moving mitochondrial science beyond function and dysfunction. Nat. Metab..

[3] Chen W., Zhao H., Li Y. (2023). Mitochondrial dynamics in health and disease: Mechanisms and potential targets. Signal Transduct. Target. Ther..

[4] Cartes-Saavedra B., Ghosh A., Hajnóczky G. (2025). The roles of mitochondria in global and local intracellular calcium signalling. Nat. Rev. Mol. Cell Biol..

[5] Suomalainen A., Nunnari J. (2024). Mitochondria at the crossroads of health and disease. Cell.

[6] Yang J., Shay C., Saba N.F., Teng Y. (2024). Cancer metabolism and carcinogenesis. Exp. Hematol. Oncol..

[7] Klemmensen M.M., Borrowman S.H., Pearce C., Pyles B., Chandra B. (2024). Mitochondrial dysfunction in neurodegenerative disorders. Neurotherapeutics.

[8] Todorova V., Blokland A. (2017). Mitochondria and Synaptic Plasticity in the Mature and Aging Nervous System. Curr. Neuropharmacol..

